# Concurrent hiatal hernia repair and gastric bypass as an adjunct in the treatment of hiatal hernia in populations with obesity

**DOI:** 10.1007/s00464-025-11854-7

**Published:** 2025-06-16

**Authors:** Leon Siegel, Rory Carroll, Dakota T. Thompson, Ryan Lehmann, Jessica Smith, Peter Nau

**Affiliations:** Department of Surgery, University of Iowa Health Care, 200 Hawkins Dr, Iowa City, IA 4628 JCP USA

**Keywords:** Gastric bypass, Hiatal hernia, Metabolic surgery, Heartburn, Regurgitation, Obesity

## Abstract

**Background:**

Minimally invasive hiatal hernia repair with fundoplication (HHR) is the standard of care for hiatal hernias but has a high risk of recurrence even in populations without obesity. Concomitant roux-en-y gastric bypass (RYGB) with HHR may mitigate the increased risk of hiatal hernia recurrence in patients with obesity while also addressing obesity-related comorbidities. There is a paucity of data on this procedure. It is hypothesized that a concomitant RYGB with HHR is safe and effective in patients with obesity.

**Methods:**

This is a single institution retrospective review of adult patients who underwent concomitant RYGB with HHR from 2014–2023. Patient charts were reviewed to collect data on complications, weight loss, GERD symptom resolution, and improvement in other obesity-related comorbidities. Outcomes were measured at one-, three-, and five-year follow-up.

**Results:**

Sixty-four patients met inclusion criteria. Fifty-three patients had primary and eleven patients had revisional surgery. There was one (2%) perioperative complication that required intervention, three (4%) unplanned readmissions for PO intolerance, and four patients (8%) treated for marginal ulcer. Resolution of heartburn/reflux symptoms was 86% at one year, 70% at 3 year, and 59% at 5 year follow-up. Improvement in diabetes (80%), hypertension (75%), and hyperlipidemia (33%) were noted at 5 years. The change in BMI and %TWL at 5 years for primary procedures was -11.5 kg/m^2^ and 37.7%, respectively. For revisional procedures, change in BMI was -2.4 kg/m^2^ and %TWL was 3.6%.

**Conclusion:**

Durability of a HHR in the setting of obesity is poor. Concomitant RYGB with HHR is safe and effective for treating GERD while also improving obesity and obesity-associated comorbidities.

A hiatal hernia is a common pathology. Its prevalence is dependent on the type of hernia with a sliding type much more common than a true paraesophageal hernia. The incidence of hiatal hernia is increasing over time and is significantly associated with obesity [1–3]. It is correlated with gastroesophageal reflux disease (GERD) and its long-term sequelae including decreased GERD-related quality of life, Barrett’s metaplasia and adenocarcinoma of the esophagus [4]. Hiatal hernia repair (HHR) is a common procedure performed at an occurrence of 19.14/100,000 persons per year [5]. The incidence of a recurrent hiatal hernia approaches 60% at five years [6]. Risk factors for a recurrence include heavy-lifting, early post-op retching, larger hernia size and obesity [7, 8]. Numerous adjuncts for decreasing this risk have been introduced, but few have provided a durable answer.

The incidence of hiatal hernias in the setting of obesity is greater than in the general population [9]. There is a direct correlation between increasing BMI and the subjective symptoms and objective severity of GERD [10, 11]. What’s more, obesity is associated with increased complications from GERD including esophageal cancer. In patients with obesity, outcomes following hiatal hernia repair are inferior compared to those of patients without obesity [12, 13]. Despite over 260,000 bariatric procedures being performed annually in the United States, there is a paucity of data on the safety and efficacy of a roux-en-y gastric bypass (RYGB) with simultaneous HHR. It is hypothesized that a concomitant RYGB with HHR is feasible, safe, and superior to a standalone HHR due to its ability to also address the patient’s obesity-associated comorbidities.

## Methods

After obtaining Institutional Review Board approval (IRB # 202,111,524), a retrospective review of a prospectively collected database from an academic tertiary referral center was completed. Inclusion criteria was any patient undergoing a RYGB for the treatment of obesity and associated comorbidities who had a planned HHR as a part of the operation. A query was performed for all cases performed from 2014 to 2023 that included primary cpt code 43,644 “laparoscopy, surgical, gastric restrictive procedure; with gastric bypass” and additional code 43,281, “repair of a paraesophageal hernia”. Charts were then reviewed out to 5 years post-operatively. Complications were recorded out to one year post-operatively and were graded using the Clavien Dindo Classification (CDC) system. Obesity-associated medical problems assessed included diabetes, hypertension, dyslipidemia and gastroesophageal reflux disease (GERD). Comorbidity improvement was defined as a decreased or cessation of medication usage. Weight loss was defined as percent of total weight loss (%TWL) at the time of the last follow-up when compared to the initial bariatric clinic appointment. Hiatal hernia-related outcomes were assessed using a standardized GERD health-related quality of life questionnaire (GERD-HRQL) at each follow-up appointment. When this was not available, narrative text was reviewed for symptoms. Frequencies and percentages were used for all categorical variables and continuous variables were expressed as means. Univariate analyses were performed using paired t-tests. Statistical analyses were performed using SPSS Version 23.0 (IBM Corp, Armonk, NY). Statistical analyses were two-sided with significance set at a *p* < 0.05.

## Results

Sixty-four patients met inclusion criteria. Fifty-three patients had a primary RYGB and HHR. Seven patients had a laparoscopic sleeve gastrectomy (LSG) converted to a RYGB with HHR. One patient had a takedown of a laparoscopic adjustable gastric band (LAGB) with HHR and RYGB (Table 1). Three patients had takedown of a fundoplication and concomitant RYGB with HHR. The mean length of stay was 1.4 days. There were no re-operations or anastomotic leaks. In one case, there was violation of the pleura intra-op necessitating a 24F chest tube placed intra-op (CDC Grade 3). Three patients (4.7%) had unplanned readmissions within the first-year for oral intolerance. One patient required IVF hydration (CDC Grade 1). In the first-year post-op there was one diagnostic EGD for epigastric pain that was unremarkable (CDC Grade 3b). Four patients were treated for marginal ulcer either empirically or based on endoscopic findings (CDC Grade 2). Of those individuals, one had documented dietary noncompliance which was the likely etiology of the marginal ulcer.

**Table 1:** Demographics and complications

Variable	Primary RYGB and HHR (*n* = 53)	Revisional HHR (*n* = 11)
Age (years, median)	48.8	53.4
Sex		
Male	3 (6.1)	1 (10)
Female	46 (93.9)	9 (90)
Pre-op mean weight (kg)	112.8	113.0
Pre-op mean BMI (kg/m^2^)	42.9	38.6
Length of Stay (days)	1.4	1.6
Revisional surgery type		
LSG to RYGB with HHR		7
LABG to RYGB with HHR		1
Fundoplication takedown and RYGB with HHR	53	3
Complications		
Leak	0 (0)	0 (0)
Readmission	2 (3.8)	1 (8.3)
Marginal ulcer treatment	4 (8)	0
Re-operation	0 (0)	0 (0)

*LABG* laparoscopic adjustable gastric band, *RYGB* laparoscopic roux-en-y gastric bypass, *LSG* laparoscopic sleeve gastrectomy, *HHR* hiatal hernia repair

Weight loss data are depicted in Table 2. The change in BMI was − 13.2 kg/m^2^ and %TWL was 30% for index procedures at one year follow-up. For those who had a bariatric revision with HHR, the change in BMI was − 5.7 and the TWL was 15%. There were thirty-three and nineteen patients available for follow-up at three and five years, respectively. The three- and five-year change in BMI for primary weight loss procedures was -11.0 and -11.5 kg/m^2^. The %TWL for primary operations was 26.2% and 37.7% at 3 and 5 years, respectively. The three- and five-year change in BMI for revisional operations was − 5.2 and − 2.4 kg/m^2^. The %TWL was 14.2% and 3.6% for 3 and 5 years, respectively.

**Table 2:** Weight loss

	One year	Three years	Five years
Primary RYGB with HHR	(*n* = 53)	(*n* = 28)	(*n* = 18)
Change in BMI (kg/m^2^)	− 13.2	− 11	− 11.5
%TWL	30	26.2	37.7
Revisional RYGB with HHR	(*n* = 11)	(*n* = 5)	(*n* = 2)
Change in BMI (kg/m^2^)	− 5.7	− 5.2	− 2.4
%TWL	15	14.2	3.6

Comorbidity resolution was documented at each time point (Table 3). Notably, resolution of symptoms associated with the hiatal hernia was 86%, 70% and 59% at one-, three- and five-year follow-up points, respectively. All patients with diabetes at enrollment were off medications or on a lower dose out to three years post-op. Of patients with hypertension, 76% had decreased medicine burden at one year, 82% at three years and 75% at five years. Thirty-one percent of patients at one year, 50% at three years and 33% at five years had improvement in dyslipidemia.

**Table 3:** Comorbidity improvement at follow-up intervals

	One year	Three years	Five years
GERD	42/49 (86%)	26/33 (70%)	10/17 (59%)
Hyperlipidemia	4/13 (31%)	6/12 (50%)	2/6 (33%)
Hypertension	25/33 (76%)	9/11 (82%)	6/8 (75%)
Type II Diabetes Mellitus	12/12 (100%)	7/7 (100%)	4/5 (80%)

Improvement is defined as either decrease in symptoms (GERD) and/or decrease in medications

## Discussion

The prevalence of obesity in the United States is 41.9%, with the medical cost of obesity-related care an estimated 173 billion in 2019 dollars [14]. Obesity is associated with several physiologic alterations in GI function that predict an increased incidence of GERD. These include increased intra-abdominal pressure, impaired gastric emptying, and decreased lower esophageal sphincter pressure [15]. Multiple studies have shown esophageal acid exposure time and reflux symptoms link positively with BMI [16–18]. Yang noted that, for each five-point increase in a patient’s BMI, the DeMeester score increases by 3 units [19]. The anatomical barriers to reflux are also more likely to breakdown in patients with obesity, which leads to a greater incidence of hiatal hernias that are symptomatic. Perhaps most concerning, this is a population that has an increased incidence of Barrett’s metaplasia and progression on to esophageal cancer when compared to a non-obese cohort [20, 21].

It has been noted that the presence of a hiatal hernia is directly correlated with the severity of GERD [22]. Recreation of the anatomical barriers to reflux with a HHR effectively addresses GERD-related complaints. Recurrence of a hiatal hernia is common, leading some to suggest that this is a progression of the disease rather than a surgical failure [23]. Hiatal hernia recurrence can be either defined as radiographic or symptomatic. While the rate of a radiographic recurrence is high, the number who present with symptoms is often lower [24]. The risk of recurrence is increased with a larger size of the original hiatal defect, early post-op retching, heavy-lifting, and obesity. The preponderance of studies investigating the risk of hiatal hernia recurrence have a mean BMI of ~ 30 kg/m^2^ [8, 24]. When considering a population with a hiatal hernia and concomitant obesity, it is difficult to extrapolate the recurrence data with confidence. With that said, the available literature suggests that obesity adversely influences surgical outcomes of a fundoplication [11, 12, 15].

As the incidence of obesity increases worldwide, surgeons will be forced to decide how to address GERD and HHR’s in this cohort. The SAGES Foregut Taskforce published a white paper advocating for the use of the RYGB to address GERD in patients suffering from concomitant obesity [15]. This is due to the RYGB’s improved durability, efficacy and ability to address a patient’s metabolic comorbidities. Notwithstanding the evidence supporting the use of the RYGB following prior gastric surgery including fundoplications, access to this procedure is frequently restricted by insurance policies that do not adhere to evidence-based medicine [25]. It is critical to populate the literature with data supporting this approach to increase patient access. To date, there is little data addressing the use of a HHR at the time of a RYGB. A retrospective review of forty elderly patients with obesity who had a paraesophageal hernia repair and RYGB showed the simultaneous procedures are both safe and effective. In this cohort, l5% of patients had a minor early complication and another 12.5% had a major complication. Notably, there was a 40% overall resolution of diabetes, and statistically significant decrease in the incidence of GERD, hypertension, sleep apnea, and dyslipidemia [26]. Another retrospective study that focused on safety and efficacy of HHR and RYGB reviewed patients over a four-year period. In their cohort of fourteen patients, five experienced perioperative complications, with two returning to the operating room within one week [27]. Others have examined performing a LSG with HHR. Lewis et al. reviewed over six-thousand patients from the Optum deidentified Clinformatics Data Mart Database, looking at LSG with and without a concurrent HHR compared to RYGB with and without HHR. At 1-year and 3-years post-surgery, the sleeve patients who had a concurrent HHR were nearly twice as likely to require an additional operation when compared to those who had a standalone LSG. Conversely, there was no difference in RYGB with or without concurrent HHR in rates of subsequent operation [28].

**Fig. 1:**
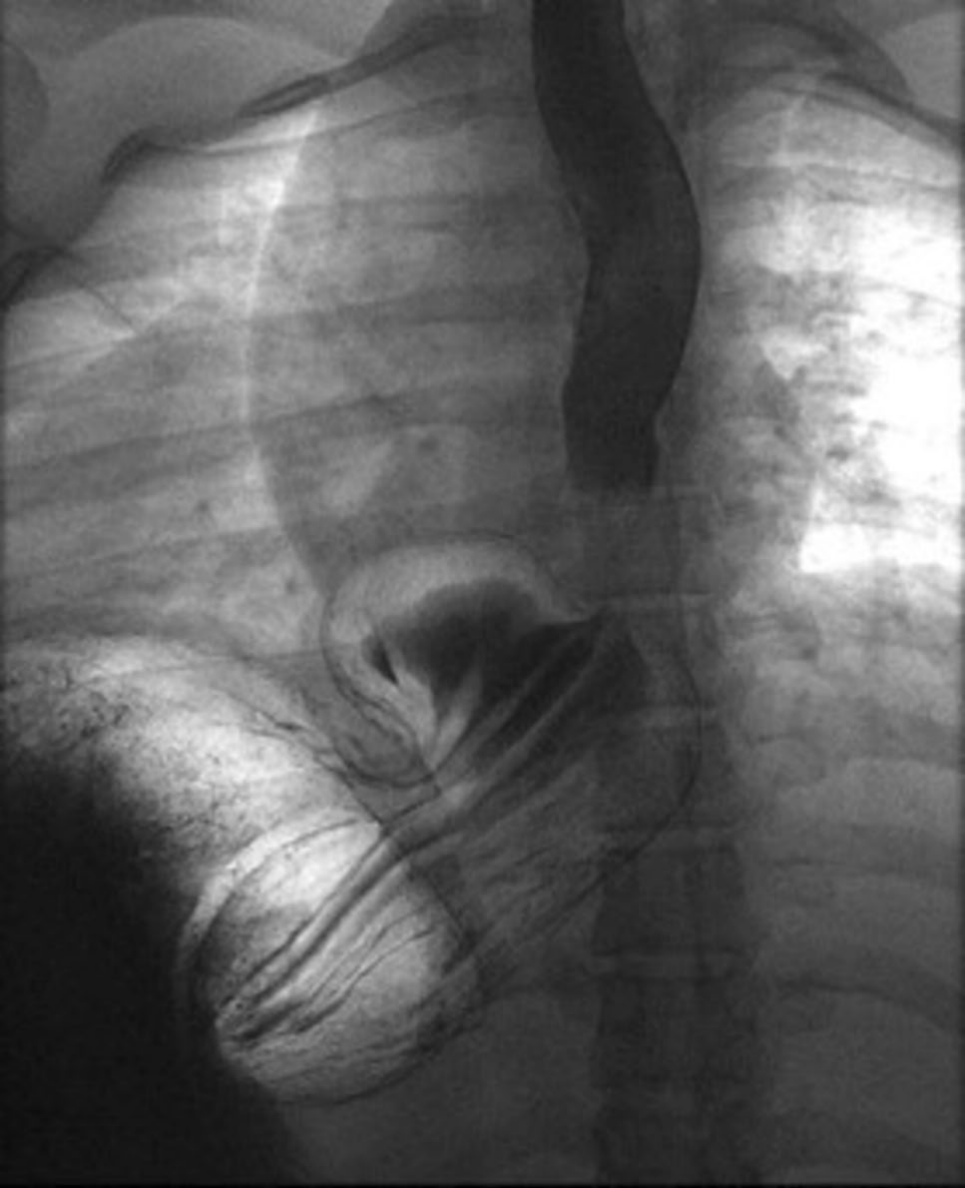
Esophagram of paraesophageal hernia addressed at the time of a RYGB

This study adds to the literature because of the inclusive nature of the population investigated and the longer follow-up time frame. The assessment of both primary and conversion operations is a more accurate depiction of contemporary bariatric surgery practice. Further, the preponderance of hernias treated were larger, type III paraesophageal with an average height of the gastroesophageal junction above the diaphragmatic hiatus greater than five centimeters (Fig. 1). In this study, the primary hypothesis was that a HHR in the setting of a RYGB was safe and effective notwithstanding prior gastroesophageal operations. The data showed that a concomitant HHR with RYGB was a safe as displayed by the infrequent and minor complications. Even in the conversion surgeries, where expected morbidity would be higher, the short-term risk was low with no difference in the mean length of stay.

A second component of the hypothesis was that a RYGB with HHR is better than a standalone HHR. In this case, improvement of obesity-related comorbidities in addition to hiatal hernia-related complaints was used as a surrogate marker for superiority, replicating the rationale used in the Foregut Taskforce’s support of the RYGB over fundoplication for GERD. In this cohort, the %TWL was excellent and in line with national averages. As anticipated, the weight loss outcomes in conversion surgeries were less than a primary bariatric procedure but was still improved over what would be seen with a HHR alone. Improvement in the obesity-related comorbidities was excellent with the preponderance of patients experiencing a de-escalation or cessation of medications. Further, subjective reports of GERD and the dependence on anti-reflux medications both decreased post-operatively. Using this definition, this data would suggest that a RYGB with HHR is superior at addressing the constellation of obesity-related comorbidities with which a patient presents inclusive of GERD rather than focusing solely on reflux-related complaints.

Limitations of this study include the retrospective nature of the data assessment with a decreasing follow-up as time from the bariatric procedure increased. The limitations of follow-up during Covid certainly played a role in this issue. Comorbidity resolution was dependent on routine follow-up with the bariatric team and primary care providers for medication de-escalation. Even with appropriate follow-up, patients did not always complete the GERD-HQRL questionnaires necessitating the use of narrative text in clinic notes to assess for the response of GERD-related symptoms. Further, it was often impossible to differentiate between the use of PPI’s for ulcer treatment versus those who had persistent GERD-related complaints. Due to this fact, the data may underestimate the degree improvement of medical problems associated with obesity. There were no routine post-operative esophagrams completed to screen for asymptomatic radiologic recurrences. Routinely obtaining esophagrams on all post-op patients would be costly as well as an inconvenience to the patient and does not coincide with the principals of value-based care. This approach more accurately reflects a real-world approach to HHR given the known risk of asymptomatic, small recurrences and the reliance on symptom improvement to define a success.

## Conclusion

Obesity is increasingly prevalent and is associated with a higher incidence of symptomatic hiatal hernias. A standalone HHR in the setting of obesity has an unacceptably high failure rate. A HHR with concomitant RYGB is feasible and safe even in the setting of prior gastric surgery.
